# An Ensemble Weighting Approach for Dendroclimatology: Drought Reconstructions for the Northeastern Tibetan Plateau

**DOI:** 10.1371/journal.pone.0086689

**Published:** 2014-01-31

**Authors:** Keyan Fang, Martin Wilmking, Nicole Davi, Feifei Zhou, Changzhi Liu

**Affiliations:** 1 Key Laboratory of Humid Subtropical Eco-Geographical Process (Ministry of Education), College of Geographical Sciences, Fujian Normal University, Fuzhou, Fujian Province, China; 2 Key Laboratory of Western China’s Environmental Systems (Ministry of Education), Research School of Arid Environment and Climate Change, Lanzhou University, Lanzhou, Gansu Province, China; 3 Department of Geosciences and Geography, University of Helsinki, Helsinki City, Helsinki, Finland; 4 Institute of Botany and Landscape Ecology, Greifswald University, Greifswald, Mecklenburg-Vorpommern, Germany; 5 Tree-Ring Lab, Lamont-Doherty Earth Observatory, Columbia University, Palisades, New York, United States of America; 6 Department of Environmental Science, William Paterson University, Wayne, New Jersey, United States of America; Chinese Academy of Sciences, China

## Abstract

Traditional detrending methods assign equal mean value to all tree-ring series for chronology developments, despite that the mean annual growth changes in different time periods. We find that the strength of a tree-ring model can be improved by giving more weights to tree-ring series that have a stronger climate signal and less weight to series that have a weaker signal. We thus present an ensemble weighting method to mitigate these potential biases and to more accurately extract the climate signals in dendroclimatology studies. This new method has been used to develop the first annual precipitation reconstruction (previous August to current July) at the Songmingyan Mountain and to recalculate the tree-ring chronology from Shenge site in Dulan area in northeastern Tibetan Plateau (TP), a marginal area of Asian summer monsoon. The ensemble weighting method explains 31.7% of instrumental variance for the reconstructions at Songmingyan Mountain and 57.3% of the instrumental variance in the Dulan area, which are higher than those developed using traditional methods. We focus on the newly introduced reconstruction at Songmingyan Mountain, which showsextremely dry (wet) epochs from 1862–1874, 1914–1933 and 1991–1999 (1882–1905). These dry/wet epochs were also found in the marginal areas of summer monsoon and the Indian subcontinent, indicating the linkages between regional hydroclimate changes and the Indian summer monsoon.

## Introduction

**Figure 1:**
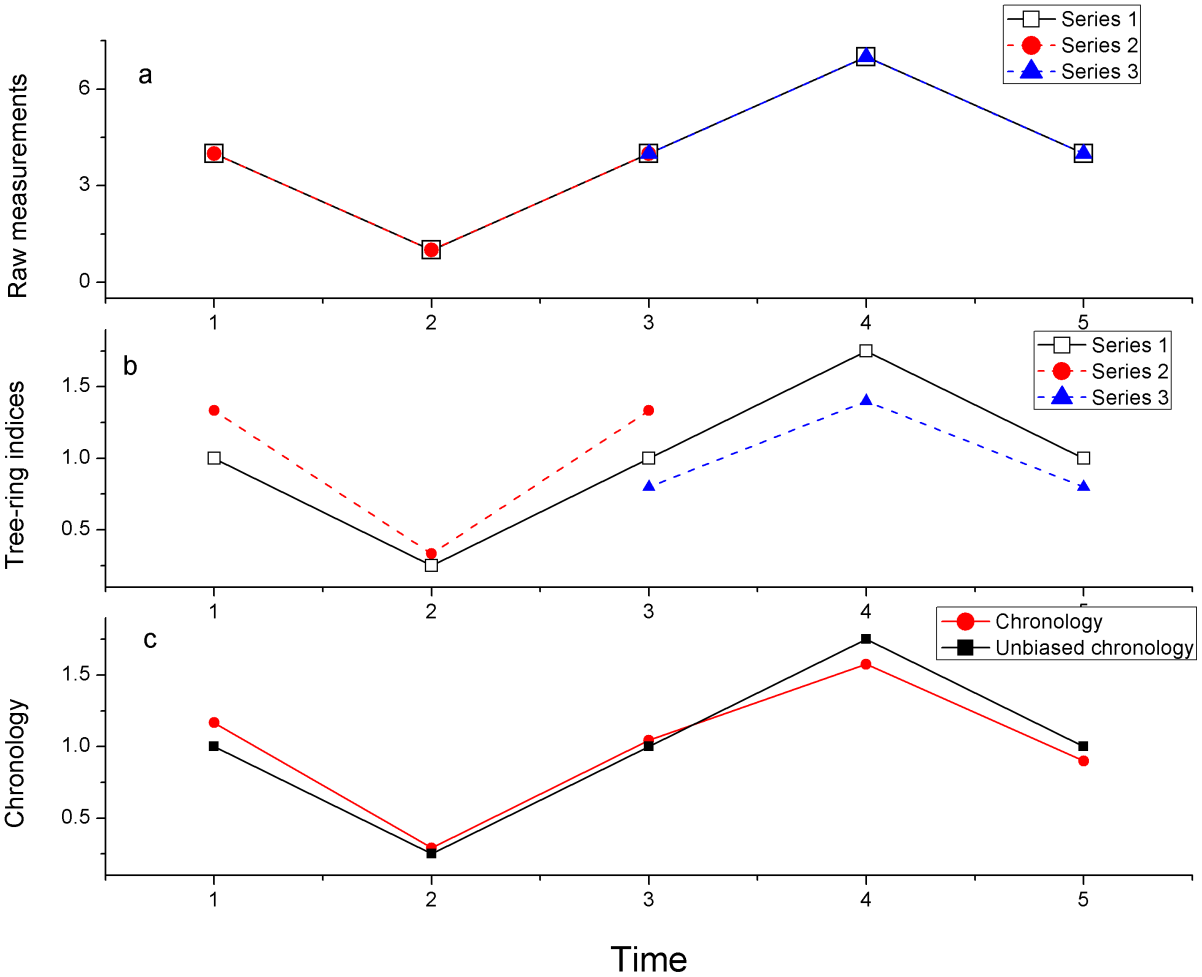
Schematic illustration of the distortion in chronology developments. (a) raw tree-ring measurements, (b) the standardized tree-ring indices calculated between the raw measurements and their mean values, and (c) the comparisons between chronology calculated as the mean among the tree-ring indices and the unbiased chronology.

Global warming has brought long-term climate data inferred from proxies into focus for both the scientific and public communities. These proxies enable us to assess recent climate change in the context of hundreds to thousands of years and to evaluate changes prior to any anthropogenic influences [1], [2]. In addition, the availability of large-scale paleoclimate data increases the robustness of the analyses of regional climate regimes in relation to external forcings and internal feedback loops [3]. Because tree-ring records are climate sensitive and can be exactly dated, they have been widely used to extend short-term instrumental climate by centuries to millennia from regional to global scales [4]. A variety of detrending methods have been developed to isolate and extract the climate signal from tree-ring series, such as negative exponential or straight line splines [5]. However, the too flexible forms result in the inevitable loss of longer-timescale climate signal. In addition, the medium-frequency (e.g. decadal/multi-decadal scales) variations can bias the final chronology [6]. This is referred to as the “trend distortion” problem, which can be mitigated by the “signal-free” method [6]. In addition, potential bias in the traditional methods can arise when setting the mean value of the tree-ring indices to 1 for different tree-ring indices covering different time intervals (Figure 1). The mean values of individual tree-ring series can be different in different temporal intervals, therefore low (high) tree-ring indices can be increased (decreased) when assigning the same value to these indices (Figure 1). Third, the tree-ring chronology can be biased when including some less climate-sensitive tree-ring series or those showing a different climate-growth relationship [7], [8]. In order to mitigate the three potential biases, we propose a method, termed the “ensemble weighting method”, to iteratively weight individual tree-ring series according to their mean climate values and by the sensitivity of each series to climate.

We use this new ensemble weighting method to develop the first tree-ring chronology from the Songmingyan Mountains in the Linxia district of the northeastern Tibetan Plateau (TP) and to ^–^ a shoulder region of Asian summer monsoon [9]. Understanding hydroclimate dynamics in regions that are only marginally affected by Asian summer monsoon is highly needed due to the sensitivity of these regions to large-scale atmospheric changes and the potential impacts of water variability. Most instrumental records from these regions begin after the 1950s and tree-ring based reconstructions are sparse, limiting our ability to understand large-scale monsoon dynamics. Although there are many climate-sensitive tree-ring chronologies available for neighboring regions in northeastern TP [10], [11], [12], [13], [14], [15], there are still no chronologies published from the Linxia district largely due to the limited availability of old-growth forests. In addition, we also test the use of this method for the development of a 1835-year tree-ring chronology in a nearby region in Dulan, which has been used for a hydroclimate reconstruction in the previous study [12], [13], [14], [15]. Therefore, the goals of this study are to (1) introduce the “ensemble weighting method”, (2) test its applicability by developing the first tree-ring chronology in the Linxia area and the tree-ring chronology in Dulan area, and (3) provide climate reconstructions for the northeastern TP and to detect its linkages with monsoon dynamics.

## Data and Methods

### The Instrumental Data

**Figure 2:**
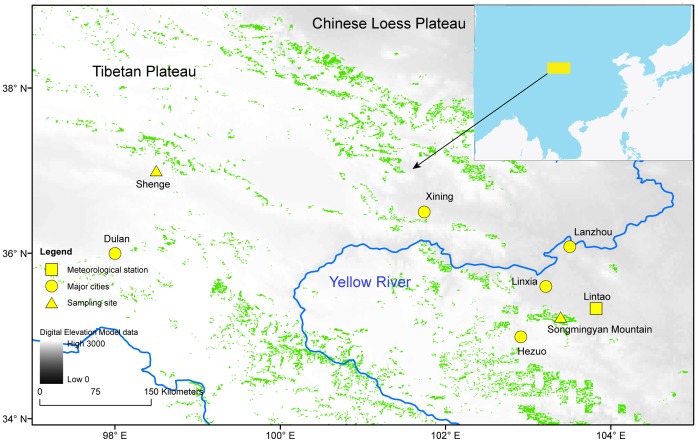
Map of the sampling site at the Songmingyan Mountain at Linxia area, the Shenge site at Dulan area, the meteorological station and major cities in the study region. The topographic features are indicated by digital elevation model data in grey colors and the boreal forests are shown in green color as well as the position of the study region in East Asia.

Monthly mean temperature peaks in July and monthly total precipitation peaks in August when the monsoon front reaches its northern shoulder region in the northeastern TP [16]. Old-growth forests in this region can only be found on the TP and on some of the mountain ranges on the arid or semi-arid Chinese Loess Plateau [17] (Figure 2). The newly introduced sampling site is located in the Songmingyan Mountain area (35.23°N, 103.39°E, 2589 m a.s.l.) in Linxia (Figure 2), in the northeastern TP. Total annual precipitation is 531 mm and annual mean temperature is 7.2°C according to the nearest meteorological station at Lintao (35.33°N, 103.82°E, 1891 m a.s.l., WMO NO. 52986). Pines and/or spruces growing near these temples are often well protected and can grow very old and can be used to develop long-term tree-ring chronologies [10], [18], [19]. The tree-ring samples used here are from Wilson’s spruce (*Picea wilsonii*) growing in the sacred green island region on Songmingyan Mountain, the biggest national forest park in the Linxia district (Figure 2). The second tree-ring site at Dulan area is located near the Shenge town, northern to the Songmingyan Mountain area with a drier and colder climate than Songmingyan Mountain [15]. Total annual precipitation is 188 mm and the mean annual temperature is 3°C in Dulan meteorological station (36.00°N, 98.00°E, 3800 m a.s.l., WMO NO. 52986).

### Tree-ring Data and Traditional Methods

We collected 30 cores from 20 *Picea wilsonii* trees at the Songmingyan Mountain site with permission from the Songmingyan Forest Park. 132 cores of 60 *Sabina przewalskii* trees were from Shenge site were downloaded from the International Tree-ring Data Bank (http://www.ncdc.noaa.gov/paleo/treering.html) [15]. These samples were mounted, air dried, polished and crossdated according to a skeleton plotting scheme by visually comparing the extremely narrow and wide rings [20]. The crossdated tree rings were measured to 0.001 mm accuracy and quality checked with moving correlations using the program COFECHA [21]. The growth trend in the raw measurements is removed by fitting an age-related growth curve (herein a cubic smoothing spline with a 50% cutoff at around 67% of the mean segment length) [22]. The dimensionless tree-ring indices are calculated as ratios between raw measurements and the fitted growth values, which are then averaged to produce a chronology based on a robust mean methodology [23]. In the signal-free method, the signal-free measurements are indexed as ratios between raw measurements and the initial robust mean chronology indices, which are again fitted with a growth curve (herein a spline) to create the “signal-free curve” representing the age-related growth trend. Then the tree-ring indices are calculated as ratios between raw measurements and the signal-free curve, which were used to create a new chronology. The final chronology is produced by iterating the aforementioned steps until the two latest versions of the chronologies showed only limited differences [6].

### Ensemble Weighting Method

**Figure 3:**
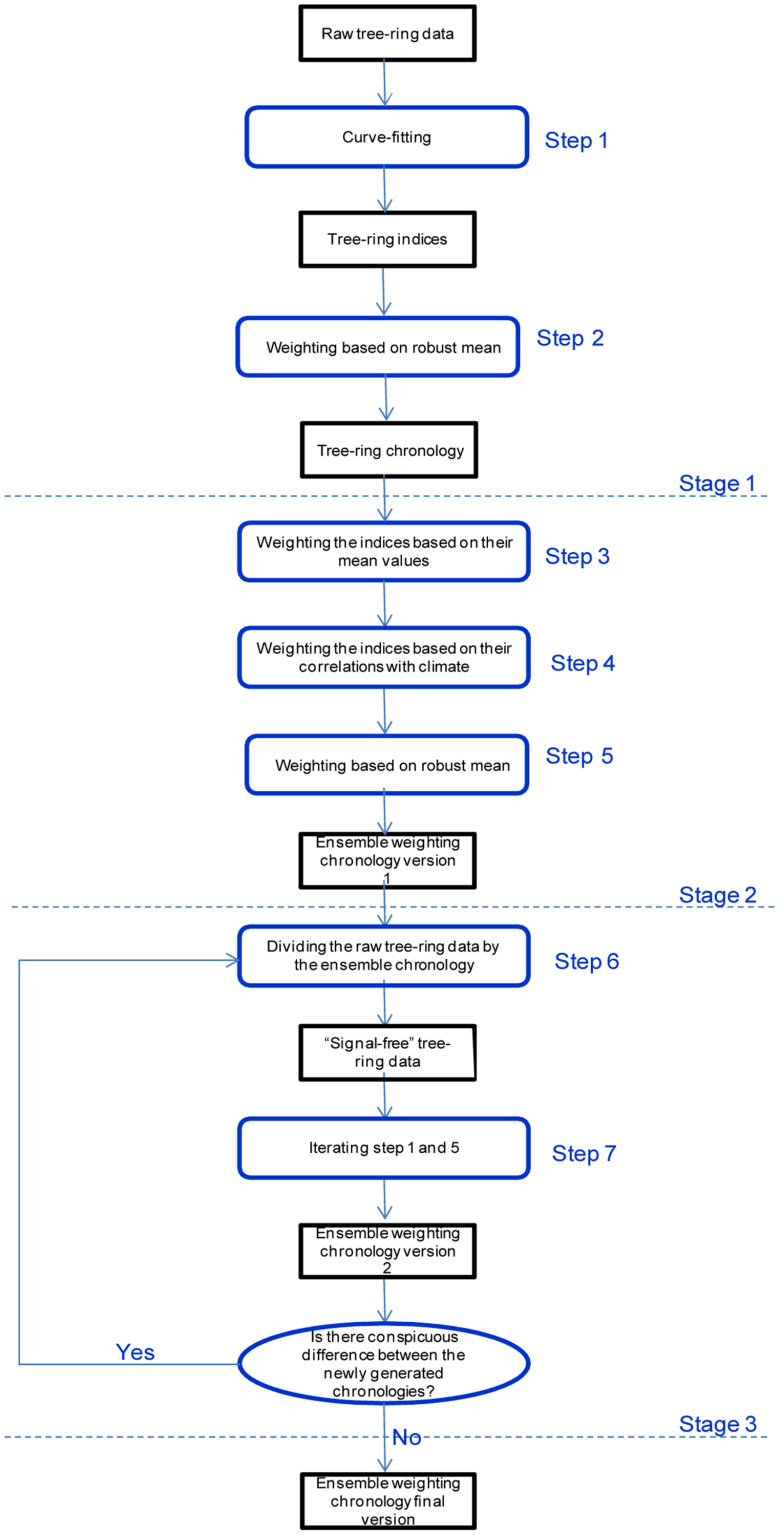
Flow diagram illustrating the development of an ensemble weighting chronology.

As shown in Figure 3, the ensemble weighting method for chronology development contains 3 stages and 6 steps, including:

**Stage 1.** Developing an initial chronology using traditional methods.

**Step 1–2.** Fitting growth curves and developing the chronologies using traditional methods detailed in the section above.

**Stage 2.** Developing the first version of the ensemble weighting chronology.

**Step 3.** The mean values of the initial chronology for the time period (segment length) the same as the duration of a given tree-ring index are calculated. These mean values are then assigned to the associated tree-ring index by multiplying the ratio between the mean chronology value corresponding to the length of the tree-ring index and the mean chronology value during the entire period.

**Step 4.** The target climate variable used for reconstruction is selected based on climate-growth relationships of the initial chronology. The Pearson correlation between resulting tree-ring indices and the target climate variable is calculated for individual tree-ring series. Each tree-ring index is weighted by its correlation with climate at different powers (herein 0, 0.5, 1, 1.5 and 2). A power of 0 equals to an unweighted series. That is,
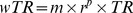
where *wTR* is the weighted tree-ring index, *TR* is the unweighted tree-ring index, *m* is the mean chronology index in the sub-period of a given tree-ring series, *r^p^* is the climate-growth correlations in different powers of *p*.

**Step 5.** The robust mean method is applied to the weighted tree-ring index to produce the chronologies (herein 5 chronologies) weighted by climate-growth correlations [8] at given powers (herein power of 5). The ensemble weighting chronology is the arithmetic mean of these chronologies, because an ensemble approach can dampen the influences of spurious climate-growth correlations due to the noise in both climate and tree ring data.

**Stage 3.** Developing the final chronology using signal-free iterations.

**Step 6.** The signal-free tree-ring measurements are produced as ratios between raw measurements and the original ensemble weighting chronology.

**Step 7.** The iteration procedures are the same as in the traditional signal-free method developed by Melvin and Briffa (2008) introduced above. The major difference from traditional applications of signal-free method is that the tree-ring indices during the iterations process are weighted. The iterations stop when only a minor difference between chronologies is found. The first and third stages, discussed above are similar to traditional methods. Major improvements occur in the second stage when the two weighting procedures are used to produce an ensemble of chronologies.

One limitation to this method, however, is the difficulty in determining the climate-growth correlations for sub-fossil samples that do not have any overlap with instrumental data. For such sub-fossil samples, we herein weight them based on their correlations with the master chronology and the correlations between the master chronology and climate, i.e. using a weighted multiplier between the series-chronology correlations and the chronology-climate correlations. The strength of the reconstructions was tested by linearly regressing the chronologies with the instrumental climate variable and evaluating the variance explained by each. The robustness of the reconstruction was further examined by split calibration-verification procedure [24], which calibrates the instrumental data from one sub-period and verifies the reconstruction using the remaining instrumental data. The verification sub-periods are the 1980–2008 and 1952–1979 for the Songmingyan Mountain reconstruction. Keeping in mind with the relatively short common period (1954–1993) between instrumental and tree-ring data at the Shenge site, we used a slightly longer sub-period for calibration (1954–1974 and 1973–1993) to maintain the robustness the split calibration-verification. Attention is also required for tree-ring series showing unstable climate-growth associations through the instrumental period [25], which could either be excluded or receive less weights. The samples used here generally show stable responses to climate through time as indicated by acceptable split calibration-verification statistics (detailed below).

## Results

**Figure 4:**
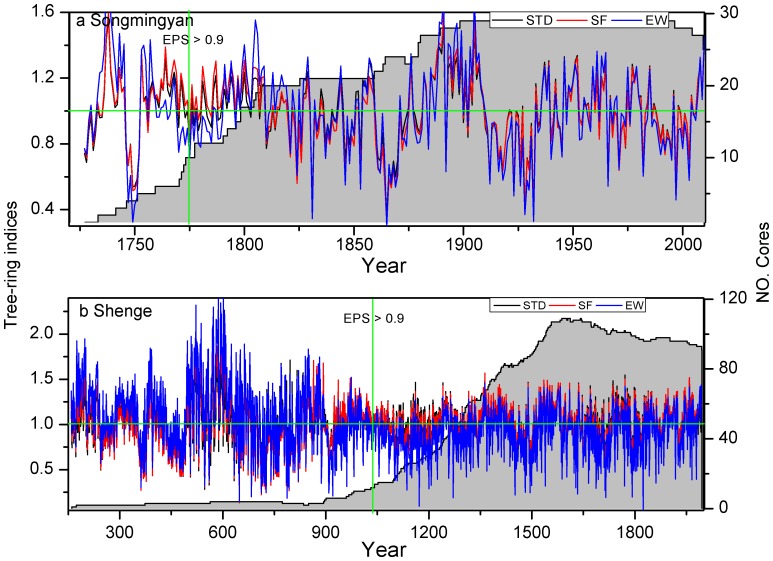
Comparisons of tree-ring chronologies based on the traditional method (STD), the signal-free (SF) method and the ensemble weighting (EW) method for (a) the Songmingyan Mountain site and (b) the Shenge site.

In this study, we applied 3 signal-free iterations for the Songmingyan Mountain site and 5 iterations for the Shenge site as suggested in previous studies [6]. We truncated the chronology at Songmingyan Mountain from 1773–2010 and Shenge chronology from 1041 to 1993 as there are sufficient replications indicated by an expressed population signal (EPS) over 0.90 [26]. Only the robust portions of the chronologies were employed in the following reconstructions. At the Songmingyan site, the signal-free method successfully mitigates the trend distortion problem by increasing (decreasing) the high (low) chronology indices in the latter half of the 18^th^ century (during the ∼1830s–1870s) (Figure 4a). At the Shenge site, the signal-free method adds back the climate signals removed in the traditional method and thus generally increase (decrease) the chronology indices when they are high (low) (Figure 4b). We additionally plotted the chronology indices derived from the three methods in the appendix (Figure S1) in a roughly 300-year interval to better illustrate the changes between them. Based on the ensemble weighting method, the chronology indices at Songmingyan Mountain increases (decreases) in the ∼1790s–1820s and near 1900s (1870s–1880s and 1900s–1950s) (Figure 4a). At the Shenge site, 4 tree-ring samples (DU07A, DU12A1, DU70A and DU70B) spanning before ∼800 show above average chronologies indices, which can be artificially lowered down in the traditional chronology. This problem has been mitigated in the ensemble weighting method that generally increases the chronology indices before ∼800 and decreases the indices afterwards (Figure 4b). Both the signal-free and ensemble weight methods are more efficient and result in larger differences during the early periods with larger variance due to the availability of only a few tree-ring samples.

**Figure 5:**
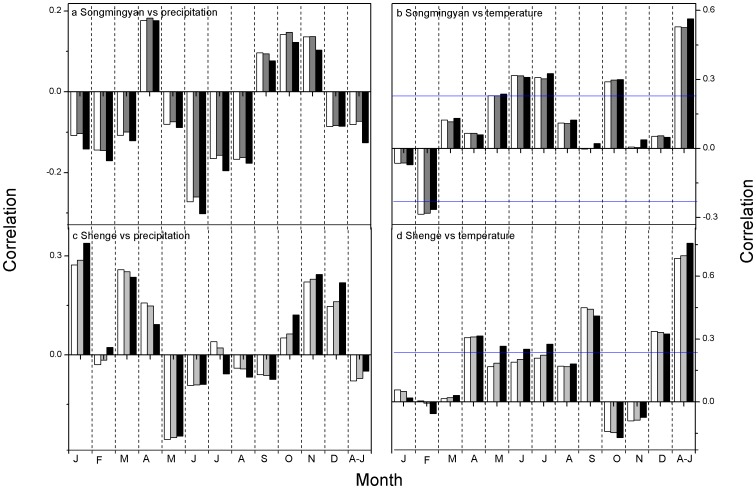
Correlations between tree-ring chronologies derived from traditional method (white bar), signal-free method (grey bar) and the ensemble weighting method (black bar) and the (a) monthly precipitation and annual precipitation from previous August to current July (A–J) for the Songmingyan Mountain, the (b) monthly temperature and annual temperature for the Songmingyan Mountain, the (c) monthly precipitation and annual precipitation from previous August to current July (A–J) for the Shenge site and the (d) monthly temperature and annual temperature for the Shenge site. The significance level of 0.1 is indicated by horizontal line.

**Figure 6:**
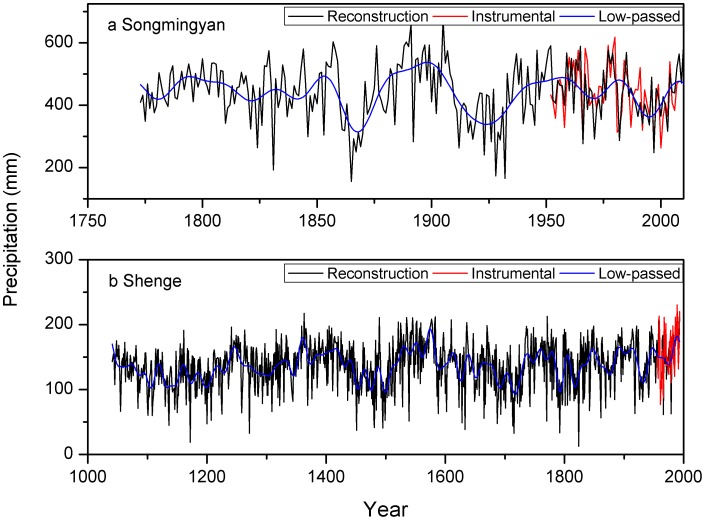
The reconstructed precipitations using the ensemble weighting chronology for the (a) Songmingyan and the (b) Shenge sites, and their low-passed values and the instrumental data.

All the chronologies show positive, significant correlations with precipitation and negative correlations with temperature in the growing season (Figure 5). The highest climate-growth correlations are found with annual precipitation from the previous August to the current July for the Songmingyan Site and from previous July to currently June for the Shenge site (Figure 5). The ensemble weighting chronology shows higher correlations with precipitation than the chronologies developed using traditional and the signal-free methods (Figure 5). Therefore our later analyses are based on the reconstruction using ensemble weighting chronology since it contains a more “pure” precipitation signal and explains a higher percent of the variance. The precipitation reconstructions derived from the ensemble weighting chronologies explain 31.7% and 57.3% of the instrumental variance for Songmingyan Mountain and the Shenge site, respectively (Figure 6). The explained variance of the Shenge reconstruction is higher than that in previous reconstruction (47.8%) using traditional methods [15].

In the split calibration-verification method, the above-zero values of reduction of error (RE) and coefficient of efficiency (CE) statistics indicate that the reconstruction model has acceptable reliability in reproducing the climate signal in both sub-periods [27]. The RE and CE are acceptable for the verification sub-periods 1980–2008 (RE = 0.38, CE = 0.19) and 1952–1979 (RE = 0.31, CE = 0.06) for the Songmingyan Mountain reconstruction. For the Shenge site, RE and CE for the verifications are acceptable for verification sub-periods of 1975–1993 (RE = 0.45, CE = 0.25) and 1954–1972 (RE = 0.73, CE = 0.68). We additionally tested the robustness of the newly introduced reconstruction at Songmingyan Mountain by comparing its extreme values with nearby reconstructions. Extremely dry years (< mean −2SD) in the reconstruction at Songmingyan Mountain are 1831, 1865, 1923, 1928, 1932 and 1997, and extremely wet (> mean +2SD) years are 1891 and 1905 (Figure 6). Some of the extreme dry years (1865, 1928 and 1997) reconstructed were also found in yearly dryness/wetness chats derived from historical documents from neighboring Lanzhou [28]. Although the extreme droughts in 1923 and 1932 were not found in the documentary records, these two extremely dry years were found in hydroclimate reconstructions in other nearby regions [10], [19], [29]. The extreme drought of 1997 was the most extreme in the Guiqing Mountain area [18], and is east of our study region.

## Discussion

### Performances of the Ensemble Weighting Method

The ensemble weighting chronologies contains two weighting procedures apart from the robust mean weighting in traditional methods [23]. This weighting procedure may have larger efficiency in modulating the chronologies for samples with both living and sub-fossil cores. This is because the living and fossil cores generally have less overlapping periods and can thus show larger differences in mean chronology values. Weighting procedure can better retain the low-frequency variations and tends to increase (decrease) the chronology indices in periods with high (low) climate signals.

Our weighting procedure is designed to put more emphasis on the most climate-sensitive samples, similar to fieldwork where scientists select sites and collect samples at sites with a high degree of climatic signal (determined by ecological conditions), at, for example, the treeline locations (a *priori* knowledge) [20]. The weighting of individual tree-ring series based on their correlations with climate provides a “quality control” to test whether individual tree-ring series contains a pure climate signal (a *posteriori* knowledge) [8]. This weighting procedure may be necessary in regions that have both arid and cold climate, such as northeastern TP. In these regions, tree growth may be sensitive to both precipitation and temperature [20] and thus it is possible to include tree-ring series with a different climate-growth relationships. In addition, this method is a more justified approach than completely excluding tree-ring samples from a chronology that have a limited climate signal. For example, the weighting of a few temperature-sensitive tree-ring series in the ensemble weighting chronology has increased (decreased) the chronology values in the warm (cold) period of the latter half of the 18^th^ century and 19^th^ century (early 19^th^ century).

This ensemble weight procedure incorporates the principles of the signal-free method that has a number of iterations to mitigate the trend distortion problem. The key difference the original signal-free method gives equal weights to the tree-ring indices. From this point of view, we can consider this ensemble weighting method as a updated version of the signal-free method. Similar to the signal-free method, our method can be improve the regional curve standardization (RCS) method, a specific technique to overcome the segment length curse problem [30]. Apart from the trend distortion problem, our method can aid in producing a climate sensitive RCS chronology. However, there is no need to adjust the mean values of individual tree-ring indices in RCS, because their mean values are the ratio between mean growth measurements and the regional growth curve and thus can be different [31], [32]. Therefore in the application of the ensemble weighting method in RCS, we only need to weight the tree-ring indices according to their associations with climate.

### Precipitation Reconstruction and Monsoon Dynamics

A drought-sensitive growth pattern, negative correlations with temperature and positive correlations with precipitation, has been widely seen in arid regions [10], [11], [12], [13], [15], [19], and is also found in our study region. Drought stress and less annual growth can occur when there are increases in evapotranspiration in warmer temperatures. We have found that tree growth at our study site correlates highest with annual averaged hydroclimate conditions rather than monthly or seasonal climate data like in our study region and some sites in southeastern TP [33], [34] and in the northeastern TP [12], [15], [35], north central China [36] and eastern Mongolia [37]. This is because tree rings can integrate monthly precipitation of consecutive months in the growing season and the precipitation from the dormant season can “compensate” for monthly water shortages [10]. This phenomenon is more conspicuous in regions with deep soil that can retain the water in winter (non-growing season) and facilitate tree growth in the following year. Tree growth at sites with very shallow soil tends to be more sensitive only to growing season hydroclimate conditions, such as the Xiaolong Mountain [10] and the Guiqing Mountain areas [18].

**Figure 7:**
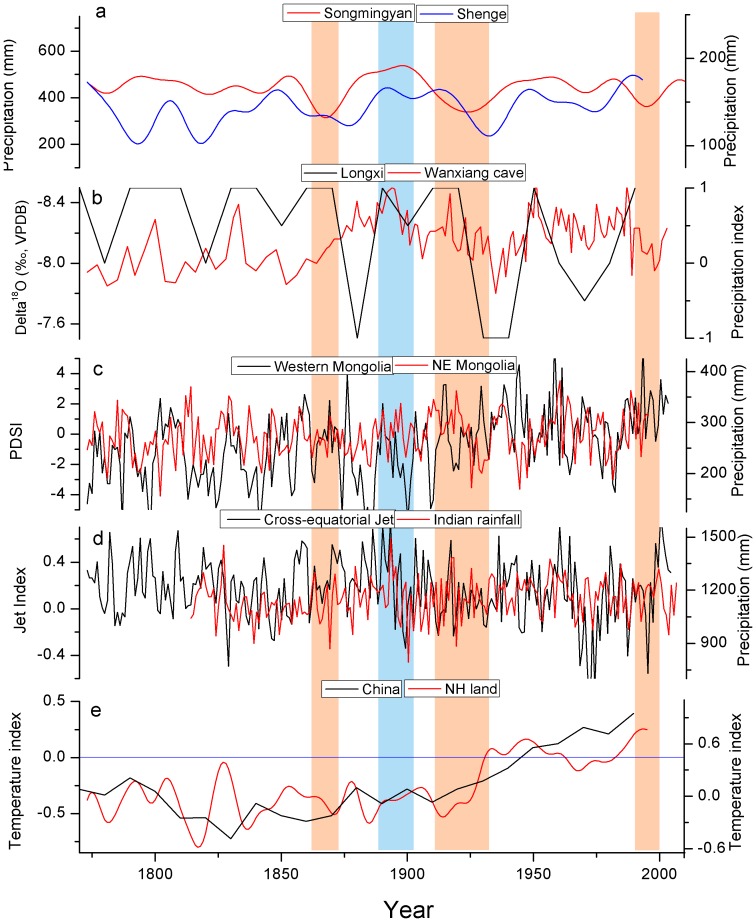
Comparisons among (a) precipitation reconstructions in the Songmingyan Mountain and the Shenge site, and (b) a speleothem-based monsoon record from the Wanxiang cave [38] southeastern to our study region and a precipitation reconstruction using historical documents from Longxi [39] eastern to our study region, (c) the hydroclimate reconstruction in far-western Mongolia [41] and the northeastern Mongolia [37], (d) coral-inferred variations of the low-level cross-equatorial jet in the western Indian Ocean [43] and the longest instrumental precipitation for all the India [42], and (e) the reconstructed temperature index in China derived from proxies of ice cores, tree rings, peat and historical documents [45] and the land temperature of northern hemisphere reconstructed from multiple proxies using composite-plus-scale method [1].

Dry epochs with more than 5 continuously dry years (< mean −SD) in the 20-year low-pass Songmingyan reconstruction are from 1862–1874, 1914–1933 and 1991–1999, and the wet epoch with over 5 continuously wet years (> mean −SD) is from 1882–1905 (Figure 7a). For the reconstruction at Shenge site, the dry epochs are found in periods of 1100–1111, 1127–1152, 1170–1178, 1192–1207, 1449–1459, 1470–1481, 1493–1506, 1686–1698, 1709–1725, 1788–1797, 1813–1822 and 1929–1935, and the wet epochs are found in the 1241–1251, 1355–1367, 1400–1421, 1525–1538, 1543–1557, 1566–1582, 1844–1851, 1888–1899, 1906–1918, 1945–1953 and 1982–1993. Our discussions only focus on the dry and wet epochs at Songmingyan Mountain, since most of these epochs have been mentioned in previous tree-ring reconstructions at Shenge site in the Dulan area [12], [13], [14], [15]. For the reconstruction at Shenge site, we herein paid special attention to the most extreme drought over the entire reconstruction period in 1824 (Figure 6b). This extreme drought was also recorded by locally historical documents the major city of Xining near the study site [28]. This drought was centered in the Dulan area in northeastern TP, which was the driest region in 1824 in the Monsoonal Asia Drought Atlas (Figure S2). The drought reconstruction for Songmingyan Mountain does not record this drought, because it is located outside of this drought center. The reconstructed precipitation dropped sharply from 100.5 mm in 1823 to 12.8 mm in 1824, and increased to 144.7 mm in 1825. Similarly, this extreme drought was not observed in 1823 and 1825 (Figure S2). This drought might indicate that an abnormally high pressure controlled the Dulan area in 1824, which requires future modeling studies to examine the occurrence of this anomalous high and its associations with large-scale circulation anomalies. Along the waveguide of westerlies, this anomalous high might be related to the abrupt shift from positive phase of Pacific/North America teleconnection to its negative phase in 1824 ().

The dry and wet epochs in the Songmingyan reconstruction are seen in hydroclimate reconstructions from neighboring regions (Figure 7a and 7b), e.g. the reconstruction from Shenge site in Dulan region and the speleothem records from Wanxiang cave [38]. However, some dry epochs in Songmingyan reconstruction, for example, in 1862–1874 and 1914–1933 are not found in a document-based precipitation reconstruction from Longxi [39], east of our study region. The Longxi reconstruction shows similar variations with the Dulan reconstruction. These differences may be related to varying hydroclimate regimes in different regions and the different seasons for reconstruction (e.g. summer or annual precipitation).

These dry/wet epochs were not only found in marginal areas of the Asian summer monsoon in the northeastern TP region, but also in northeastern China and northeastern Mongolia, such as the dry epoch from 1914–1933 [10], [18], [29], [37], [38], [40] (Figure 7c). Our study adds additional proxy evidence that this dry epoch reached the southwestern boundary of the Songmingyan Mountain. In the northeastern TP region this drought began in the 1910s, nearly a decade before other regions, including north central China and eastern Mongolia, suggesting that this persistent drought initiated from the west. However, the hydroclimate variations have limited resemblance with the hydroclimate changes from the westerlies-dominated regions, such as in western Mongolia [41] (Figure 7c). This indicates that hydroclimate changes in the past two centuries are more likely dominated by the monsoon and are different from the hydroclimate changes in westerlies-dominated regions, which is in agreement with previous studies [9]. Additional evidence of the influences of the monsoon in this region comes from the similar variations between hydroclimate changes and a long instrumental precipitation record from India [42] and the coral-inferred low-level cross-equatorial jet in the western Indian Ocean [43] (Figure 7d). Previous studies documented that the Asian summer monsoon, particularly the Indian summer monsoon, can reach northeastern TP along the eastern boundary of TP [44]. The dryness (wetness) in the northeastern TP and associated weakened (strengthened) Indian summer monsoon often corresponds to cold (warm) periods of the reconstructed land temperature of the northern hemisphere [10] and China [45], except for the recent monsoon failure since around the 1980s (Figure 7e). The positive relationship between temperature and the monsoon is likely a result of increased land-ocean temperature gradients [9], [38] and the northward shifts of the intertropical convergence zone (ITCZ) [46]. The monsoon failure in recent decades may be caused by intensified human activities such as increases in aerosols emissions, which can weaken the land-ocean temperature gradients and the gradients between the northern and southern hemispheres [47] and the cooling of the upper troposphere [48].

## Conclusions

We introduced an ensemble weighting method to alleviate two potential biases in traditional methods of chronology development. This method allows the mean value of tree-ring series to vary at different time intervals, instead of assigning a value of 1. In addition, this ensemble weighting method assigns weights to individual series depending on the strength of the climate-growth relationship. The resulting chronology is then averaged from an ensemble of chronologies with weighted individual tree-ring indices. The chronology development is iterated to adjust the mean values of individual tree-ring indices and to alleviate the trend distortion problem, similar to signal-free methods. We tested the efficiency of this method by developing a new tree-ring chronology at the Songmingyan Mountain and by recalculating a tree-ring chronology at Shenge site from a marginal area of the Asian summer monsoon in the northeastern TP. These reconstructions explain higher instrumental variance, 31.7% for the Songmingyan reconstruction and 57.3% for the Shenge reconstruction, than the reconstructions based on traditional methods. The reconstructed dry epochs range from the marginal area of the Asian summer monsoon from the northeastern TP to eastern Mongolia, as well as the monsoon dominated Indian subcontinent, indicating the linkages between regional hydroclimate changes and the Asian summer monsoon.

## Supporting Information

Figure S1 Indices of the tree-ring chronologies developed from traditional method (black), the signal-free method (red) and the ensemble weighting method (blue) for the Shenge site at a roughly 300-year interval.
(TIF)

Figure S2 The reconstructed summer (June-August) Palmer Drought Severity Indices in years of 1823, 1824 and 1825 from the Monsoon Asia Drought Atlas (Cook et al. 2010).
(TIF)
