# Supplementary figures and images for: An Ensemble Weighting Approach for Dendroclimatology: Drought Reconstructions for the Northeastern Tibetan Plateau
Source: PLoS One. 2014 Jan 31;9(1):e86689. doi: 10.1371/journal.pone.0086689 (PMC3908956; doi:10.1371/journal.pone.0086689)

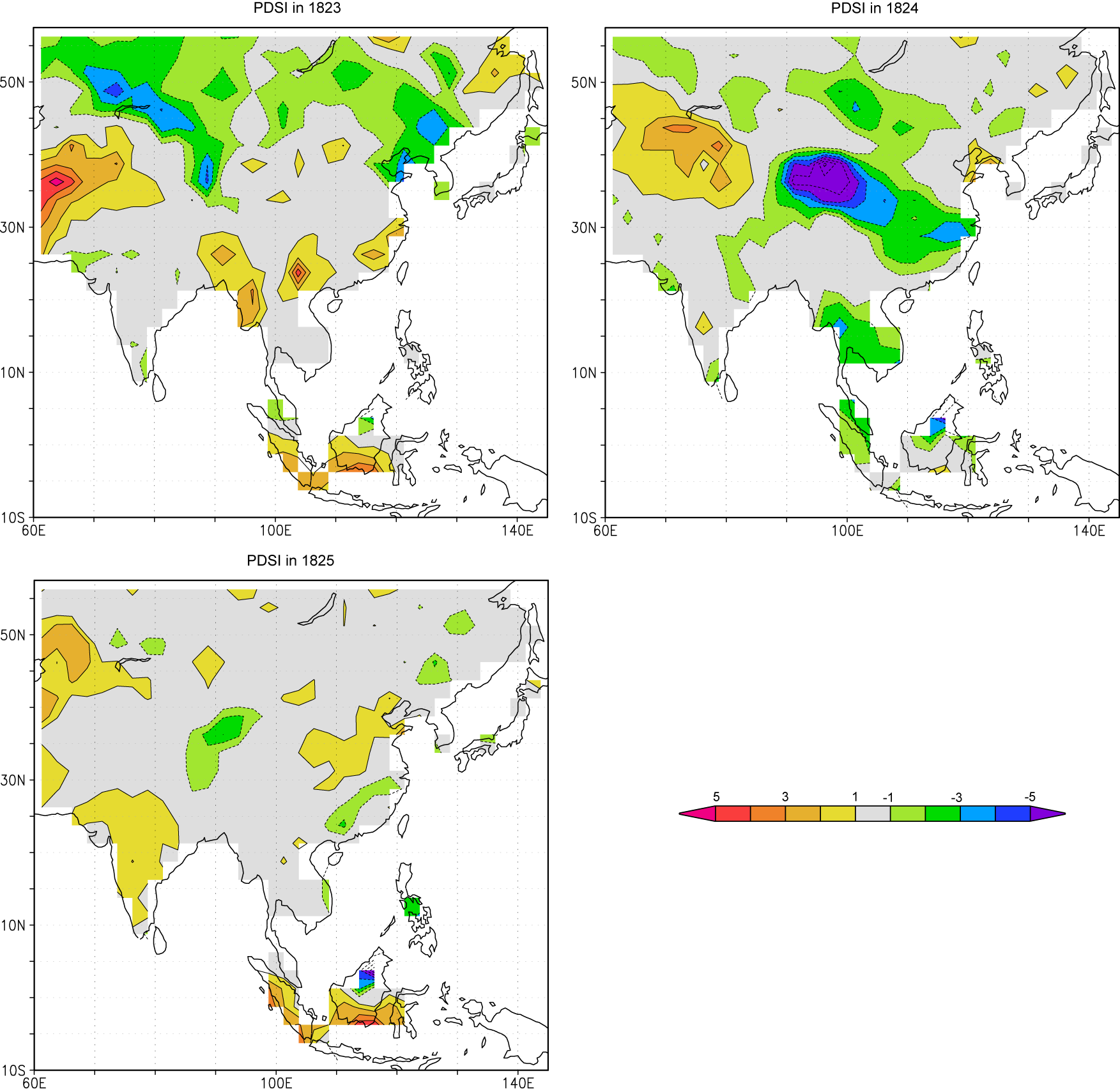

Supplement: Figure S2 — The reconstructed summer (June-August) Palmer Drought Severity Indices in years of 1823, 1824 and 1825 from the Monsoon Asia Drought Atlas (Cook et al. 2010). (TIF) [file pone.0086689.s002.tif]
